# Explainable AI analysis of brake control in CARLA through reference and distilled policies

**DOI:** 10.3389/frobt.2026.1847808

**Published:** 2026-08-03

**Authors:** Chenghao Wang, Lingyun Ke, Xiaoming Liu, Danni Huang, Jingyu Yao, Weishen Chu

**Affiliations:** 1 College of Computing, Georgia Institute of Technology, Atlanta, GA, United States; 2 Department of Electrical Engineering and Computer Science, Syracuse University, Syracuse, NY, United States; 3 Department of Computer Science, San Jose State University, San Jose, CA, United States; 4 School of Engineering and Computing, Trine University, Angola, IN, United States; 5 Department of Computer Science, Yale University, New Haven, CT, United States; 6 Department of Electrical and Computer Engineering, Northwestern University, Evanston, IL, United States

**Keywords:** brake control, CARLA simulation, explainable AI, policy distillation, shap, surrogate modeling

## Abstract

This paper presents an explainable AI analysis of brake control in CARLA closed-loop driving. Longitudinal braking is studied through a threshold policy, a risk-aware reference policy, and a distilled policy learned from reference rollouts. The framework combines structured traffic-state features, high-fidelity XGBoost surrogates, and SHAP to analyze deployed brake behavior across Town10HD and Town05. Results show that brake generation is consistently dominated by front-vehicle distance, relative speed, and time to collision, while lateral variables contribute weakly. The distilled controller retains a forward-risk-oriented explanation structure rather than behaving as an arbitrary black box, but the degree of apparent semantic alignment with the reference policy is environment-dependent. Fallback-aware analysis shows that this alignment is substantially reinforced by the deployed safety fallback in Town10HD, while Town05 provides a cleaner view of the learned component itself. These findings show that learned brake control can remain interpretable when grounded in semantically structured state representations.

## 1 Introduction

Learning-based control has become increasingly important in robotics and autonomous driving, especially in environments where uncertainty, interaction, and nonlinear system behavior limit the effectiveness of purely fixed control rules. At the same time, the deployment of learning-enabled controllers in real-world systems remains strongly constrained by safety requirements, since control outputs directly influence physical behavior and can lead to hazardous outcomes. In autonomous driving, this challenge is further amplified by the limited interpretability of many learned decision mechanisms, which makes them harder to inspect, diagnose, and justify in safety-critical settings. Consequently, the practical adoption of learned control policies depends not only on predictive or control performance, but also on transparency and explainability Brunke et al. (2022); Kiran et al. (2022); Zablocki et al. (2022).

Among the different control channels in autonomous driving, braking is particularly important because it is tightly coupled with immediate safety risk. A braking action must react to forward spacing, relative motion, and collision risk in a timely and consistent way, and it must remain understandable enough for debugging, comparison, and behavioral analysis. However, many existing studies discuss autonomous driving systems at the level of overall driving success, trajectory quality, or end-to-end performance, which can obscure the specific decision structure behind a single safety-critical action. This creates a gap between high-level evaluation and action-level interpretability, especially for braking, where the key question is not only whether the vehicle avoided failure, but also why braking was triggered and which factors dominated the decision Zablocki et al. (2022); Kiran et al. (2022).

To address this problem, this paper studies brake generation as a focused interpretability problem rather than attempting to explain a full end-to-end driving stack. The proposed framework is implemented in CARLA, an open urban driving simulator developed to support the training, development, and validation of autonomous driving systems. Within this closed-loop environment, the controller adopts a decoupled architecture: lateral steering is handled by a bicycle-model-based MPC module, while longitudinal brake generation is governed by one of three policies, namely, a threshold-based brake policy, a risk-aware reference policy, and a distilled brake policy learned from reference policy rollouts. This design makes it possible to isolate braking as the primary object of analysis while preserving realistic traffic interaction during deployment Dosovitskiy et al. (2017).

The threshold-based brake policy serves as the most interpretable baseline. It relies on explicit triggers defined over forward safety quantities such as front vehicle distance, time to collision, and relative speed. The risk-aware reference policy extends this logic by transforming several safety cues into continuous risk components and combining them into a graded brake output. The distilled brake policy is then trained as a structured approximation of the reference policy using rollout data collected in CARLA. Importantly, the distilled policy operates on semantically meaningful traffic state features rather than raw sensory observations. At the same time, the deployed distilled controller is hybrid rather than purely learned, because it includes an explicit safety fallback under predefined risky conditions. This makes it a useful setting for examining not only whether a learned braking policy retains a forward-risk-oriented explanation structure after policy distillation, but also how much of the apparent alignment with the reference policy is preserved by the learned component itself and how much is reinforced by the deployed fallback mechanism.

To explain the deployed brake behavior of each policy, the paper introduces a surrogate-based interpretability pipeline. For each policy and each town, closed-loop rollout logs are collected, a high-fidelity XGBoost surrogate model is trained to reproduce realized brake outputs, and SHAP is then used to analyze global feature importance, feature directionality, and local decision attribution. XGBoost provides an effective tree boosting framework for structured regression problems, while SHAP offers a unified feature attribution approach for interpreting model predictions Chen and Guestrin (2016); Lundberg and Lee (2017). In addition to global, braking-only, and nonzero time-to-collision analyses, fallback-aware analysis is introduced for the distilled controller to separate the learned component from the deployed hybrid safety behavior. Quantitative similarity measures are further used to characterize the degree of explanation-structure alignment between the reference and distilled policies.

The central finding of the paper is that braking decisions are consistently governed by a compact set of forward-risk variables, especially front vehicle distance, time to collision, and front vehicle relative speed, while lateral tracking variables contribute comparatively little. More importantly, the distilled controller retains a forward-risk-oriented explanation structure rather than behaving like an arbitrary black box, but the degree of apparent semantic alignment with the reference policy is environment-dependent. In Town10HD, the deployed explanation structure is strongly influenced by fallback activation, whereas Town05 provides a cleaner view of the learned component itself. These findings suggest that, under structured policy design and closed-loop analysis, learned braking behavior can remain substantially more interpretable than a fully opaque control policy.

The main contributions of this work are fourfold. First, the paper formulates braking as a focused control interpretability problem in closed-loop autonomous driving. Second, it introduces a comparative hierarchy of threshold, reference, and distilled brake policies within the same simulation framework. Third, it proposes a surrogate-plus-SHAP pipeline for interpreting deployed brake behavior rather than only offline model outputs. Fourth, it introduces fallback-aware and quantitative explanation analyses to characterize how much of the apparent alignment between the reference and distilled policies is preserved by the learned component and how much is reinforced by the deployed safety fallback across different simulation environments.

## 2 Related work

### 2.1 Braking control and automatic emergency braking

Braking has long been recognized as one of the most safety-critical functions in intelligent vehicles. A substantial body of work on automatic emergency braking (AEB) and collision mitigation has focused on when braking should begin, how aggressively braking should be applied, and how braking performance changes under different traffic and road conditions. Time-to-collision (TTC) remains one of the most widely used ideas in this literature. Recent AEB work continues to rely on TTC thresholds and braking-pattern design to improve rear-end crash avoidance while also accounting for ride comfort Lai et al. (2024), and counterfactual studies of real crash data have shown that AEB can be highly effective while still degrading under higher speeds or poor weather conditions Seacrist et al. (2020).

Beyond simple TTC triggering, more advanced emergency braking and collision-avoidance methods increasingly incorporate multiple safety indicators and surrounding-vehicle behavior. Zhao et al. (2023) proposed a collision-free emergency braking system that considers surrounding vehicle intentions during dangerous maneuvers, while Nguyen et al. Nguyen et al. (2023) developed risk-informed decision-making and control strategies that explicitly evaluate TTC and DRAC in emergency situations. Li et al. Li et al. (2021) likewise proposed a probabilistic risk-assessment approach for collision avoidance in multiple scenarios, using multiple safety indicators and adjustable driving styles. Taken together, these studies show that braking and emergency response are already moving from rigid threshold logic toward richer multi-factor risk reasoning.

However, most of this line of work is still optimized for safety performance, comfort, path feasibility, or crash reduction, rather than for interpretability of the deployed brake decision itself. In other words, these methods are often evaluated by whether they brake effectively, not by whether the brake mechanism can be structurally explained in closed loop. That gap is especially important in a safety-critical setting, where understanding why braking occurred may be nearly as important as showing that braking improved safety.

### 2.2 Risk-aware decision making and safe learning for autonomous driving

A second closely related line of work concerns risk-aware decision making and safe learning for autonomous vehicles. Recent reviews note that reinforcement learning has become an important paradigm for autonomous-driving behavior planning and control, with tasks such as car following and decision making under interaction appearing frequently in the literature Wu et al. (2024). At the same time, these reviews also emphasize persistent challenges around safety, generalization, and reliable real-world deployment. More specifically, safe reinforcement learning reviews for autonomous-vehicle control highlight that unconstrained RL can explore unsafe actions and therefore requires explicit risk estimation, constraints, or safety filters Inamdar et al. (2024). More broadly, safe learning in robotics has been framed as the challenge of achieving improved decision making under uncertainty while respecting safety constraints Brunke et al. (2022).

This concern also appears in braking-specific learning work. Chae et al. (2017) proposed a deep-reinforcement-learning-based autonomous braking system for pedestrian scenarios, explicitly arguing that conventional autonomous braking systems are often rule based and hard to adapt to diverse uncertainty, while learned braking can better balance accident avoidance and behavioral timing. Nevertheless, most risk-aware and safe-learning studies still evaluate policies through success rate, reward, collision reduction, or constraint satisfaction. They do not usually ask a narrower interpretability question such as which variables dominate brake generation in deployment, how a structured risk policy differs from a learned one, and which semantic elements survive policy approximation or distillation.

### 2.3 Explainability in autonomous driving

Explainability in autonomous driving has become a major research topic, largely because highly capable AI-based vehicle systems remain difficult to inspect and justify in safety-critical situations. Omeiza et al. (2021) review explanations across multiple autonomous-driving operations, including perception, localisation, planning, control, and system management, and emphasize that AVs should be able to explain what they have seen, done, and might do. Zablocki et al. (2022) specifically review explainability for deep vision-based autonomous driving systems trained with behavior cloning, while Dong et al. (2023) study explainable autonomous driving through image-based language generation to improve trustworthiness of AI driving decisions. A more recent systematic review on explainable AI for safe and trustworthy autonomous driving frames XAI contributions in terms of interpretable design, surrogate models, monitoring, auxiliary explanations, and validation Kuznietsov et al. (2024).

Recent work outside autonomous driving also helps clarify the broader methodological role of XAI. Practical reviews have framed the transition from opaque AI systems to more transparent and usable “glass-box” reasoning as a central challenge for real deployment Liu X. et al. (2025). Related trustworthy-AI discussions have appeared in high-stakes communication and governance settings, including explainable AI for 5G security and privacy Liao et al. (2025) and explainable semantic anomaly detection in 10-K risk disclosures Sun et al. (2026). These studies are not braking-control papers, but they reinforce an important point: interpretability is increasingly treated as a deployment requirement in domains where trust, auditability, and operational accountability matter.

Taken together, these works make two things clear. First, explainability is now widely recognized as necessary for trustworthy autonomous driving. Second, a substantial share of the literature has emphasized perception-heavy or end-to-end explainability problems, especially vision-based self-driving and explanation interfaces for complex AI systems. There has been relatively less emphasis on tightly scoped, low-level action channels such as brake generation under structured closed-loop control. Even when control is included in surveys, it is usually one category among many, rather than the main explanatory object.

### 2.4 Explainability, structured reasoning, and trustworthy AI in broader domains

A related observation is that many recent AI systems in vision, document intelligence, and policy understanding increasingly operate in settings where robustness, structure, and transparency are all important, even when the primary contribution is not framed as XAI. For example, recent person re-identification studies have explored camera-aware graph consistency and local feature enhancement to improve robustness across challenging visual domains (Liu et al., 2025b; Liu et al., 2025c). In document intelligence, multi-hop retrieval-augmented generation has been studied for document-level question answering with large language models Huang et al. (2025a), including financial and compliance contexts where traceability and multi-step reasoning are especially important Huang et al. (2025b). Structured reasoning also appears in privacy-related language understanding, where multi-granularity adapter fusion has been proposed for structured privacy policy interpretation Yu et al. (2025).

These works should not be conflated with autonomous-driving control or with surrogate-based explanation of learned policies. However, they do help motivate the broader context of the present study. Across vision, document analysis, privacy, communications, and financial compliance, modern AI systems are increasingly expected not only to perform well, but also to support structured reasoning, traceability, and user trust. The present paper contributes to this broader trend by examining whether brake behavior in closed-loop driving can remain interpretable after policy learning and policy distillation.

### 2.5 Explainability in robotics and control

Related concerns also appear in robotics more broadly. Setchi et al. (2020) introduced the notion of “Explainable Robotics,” arguing that explanations are necessary for trustworthy human-robot interaction and that different users require different forms of explanation depending on the autonomy level and interaction context. Recent robotics work on explainability of deep reinforcement learning similarly argues that the problem is even more acute in robotics because learned policies operate in the physical world and often cohabit space with humans. For example, Taghian et al. (2024) interpret robotic DRL policies through graph-based representations and layer-wise relevance propagation, showing that action-level policy interpretation is possible but still methodologically demanding.

At the same time, broader robot safety surveys continue to identify safety control as a fundamental bottleneck for real deployment, especially in unstructured or human-facing environments Zhang et al. (2025). This reinforces a key premise of the present paper: in robotics and autonomous driving, the need for explanation is not merely philosophical or regulatory; it is operational. A learned policy that cannot be meaningfully inspected is harder to debug, harder to validate, and harder to trust when physical risk is involved.

### 2.6 Interpretable policies, distillation, and surrogate explanations

A final relevant line of research concerns interpretable policies and policy simplification. Hein et al. (2018) showed that interpretable reinforcement-learning policies can be learned as explicit equations using genetic programming, and argued that understandable policies are more likely to be deployed in industrial settings. More recently, Xing et al. (2023) proposed to improve the interpretability of RL policies through policy distillation and selective input-gradient regularization, including experiments in CARLA autonomous driving. These works are highly relevant because they suggest that policy distillation can be used not only for compression or transfer, but also as a route toward more interpretable behavior.

In parallel, explainable autonomous-driving reviews increasingly identify surrogate models as an important mechanism for generating interpretable explanations for otherwise opaque predictors. The recent systematic review on XAI for safe and trustworthy autonomous driving explicitly lists interpretable surrogate models as one of the major contribution classes of XAI in autonomous driving Kuznietsov et al. (2024). However, the existing literature still leaves an important gap. Prior distillation and interpretable-policy work typically studies general RL policy behavior or saliency efficiency, not the preservation of braking semantics under structured reference-to-distilled policy learning. Likewise, surrogate-based XAI in autonomous driving is usually discussed at a general architectural level rather than being used to compare threshold-based braking, risk-aware reference braking, and a distilled braking policy within the same closed-loop framework.

### 2.7 Position of this work

In summary, existing braking-control research provides strong foundations for TTC-based braking, risk-aware emergency control, and safety-oriented collision avoidance, but usually emphasizes performance rather than interpretability. XAI research in autonomous driving has established the importance of explanations, yet much of it has focused on perception-heavy or end-to-end systems instead of low-level brake behavior. Broader XAI and trustworthy-AI work in communications, compliance analysis, privacy understanding, document intelligence, and robust visual recognition further reinforces the value of structured, inspectable AI behavior in high-stakes settings. Explainable robotics and interpretable-policy studies show that learned policies can in principle be made more understandable, but they stop short of analyzing how a structured safety policy’s semantic content is preserved in a deployed braking controller. The present paper is designed around exactly this missing connection. It studies brake generation as the central explanatory object, compares threshold, reference, and distilled policies within the same CARLA closed-loop framework, and uses surrogate plus SHAP analysis to characterize which semantic risk factors remain dominant after policy distillation.

## 3 Methods

### 3.1 Closed-loop system overview

The proposed framework is implemented in CARLA as a closed-loop driving system in which the ego vehicle repeatedly senses the current road and traffic state, computes steering and braking commands, applies the commands online, and logs the resulting trajectory and control behavior for subsequent analysis. The architecture is intentionally decoupled: lateral tracking is handled by a model predictive controller, whereas longitudinal brake generation is governed by an explicit brake-policy module. This separation allows the present study to isolate brake generation as the primary object of explanation while still preserving realistic traffic interaction and control feedback during deployment.

**FIGURE 1:**
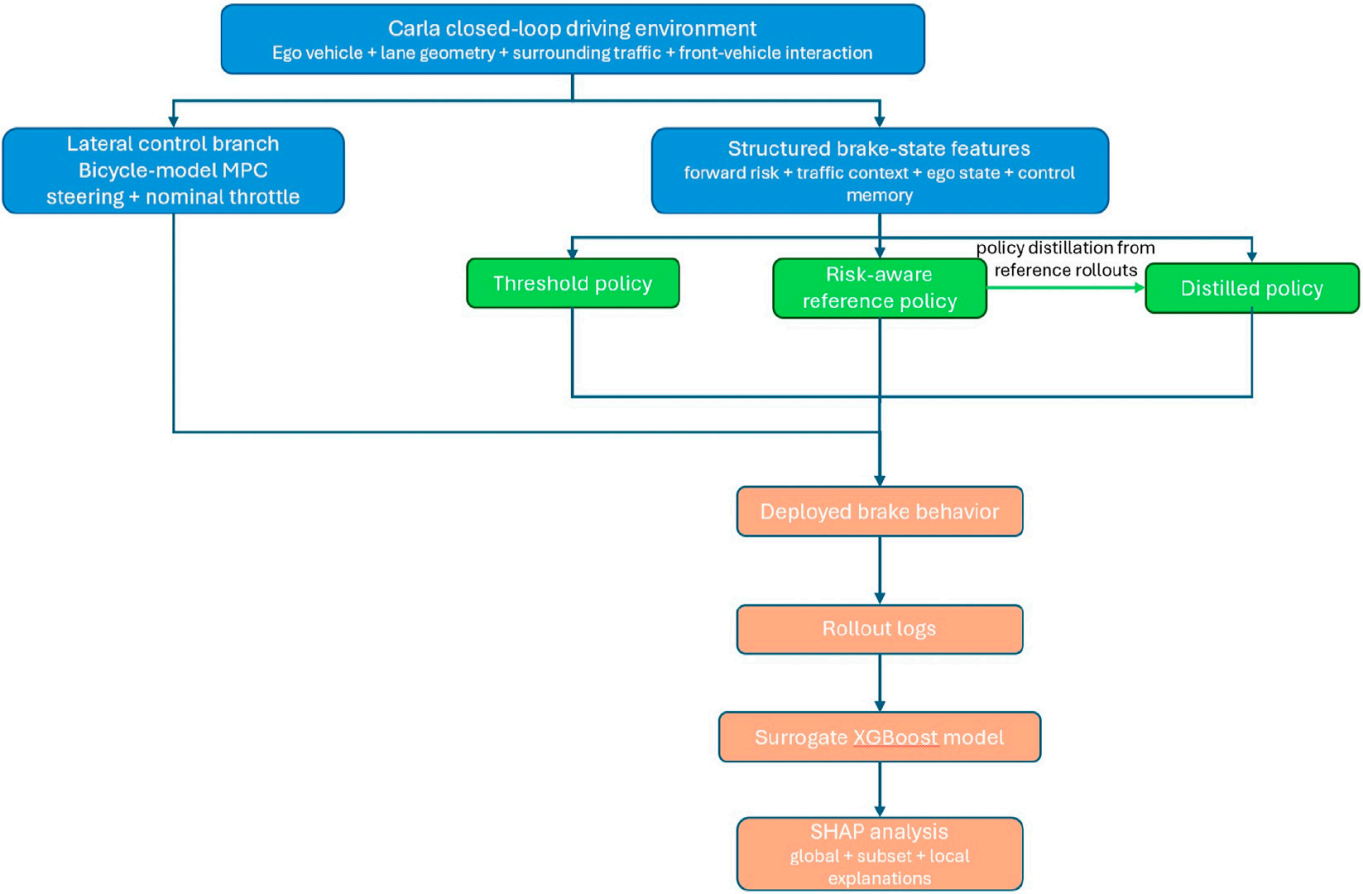
Overall framework of the proposed explainable brake-control analysis in CARLA. A decoupled closed-loop architecture is used, where lateral tracking is handled by bicycle-model MPC and braking is governed by threshold, reference, or distilled policies. The distilled policy is learned from reference-policy rollouts using structured brake-state features. Deployed brake behavior is logged, approximated by surrogate XGBoost models, and interpreted through SHAP analysis.

Figure 1 provides an overview of the proposed closed-loop brake-control and interpretability framework.

At each control step, the simulator provides the ego pose, velocity, lane geometry, and surrounding traffic states. The controller then computes lateral tracking errors relative to the current lane reference, extracts traffic interaction features such as front-vehicle distance, relative speed, and time to collision, and applies a steering command from MPC together with a brake command from one of the evaluated brake policies. The executed command and the resulting state transition are written into a structured rollout log through the MPCRecordschema. This log serves as the common data source for policy distillation, surrogate training, and SHAP-based interpretability analysis.

### 3.2 Lateral tracking controller

Although the paper focuses on braking, lateral control provides the closed-loop context in which braking behavior is expressed. The main steering controller is a simplified bicycle-model MPC defined on error states *x* = [*e*_*y*_ *, e*_*ψ*_]^⊤^, where *e*_*y*_ denotes lateral error and *e*_*ψ*_ denotes heading error. The control input is steering angle *δ*. The controller uses a discrete linearized error-state model of the form

$$e_{y}\;\left(k+1\right)=e_{y}\left(k\right)+v\;e_{\psi }\left(k\right)\Delta t$$

$$e_{\psi }\left(k+1\right)=e_{\psi }\left(k\right)+\frac{v}{L}\;\delta \left(k\right)\Delta t-v\kappa_{ref}\Delta t$$

where v is the ego speed, L is the wheelbase, and κref is the reference curvature estimated by averaging the local curvature and lookahead curvature. In implementation, the controller uses Δ*t* = 0.1 s, horizon length *N* = 12, wheelbase *L* = 2.8 m, and steering bounds *|δ| ≤* 0.6. The steering-rate constraint is implemented through *|δ*_*k*_ *− δ*_*k*−1_ *| ≤* 0.12 or 0.14, depending on the script variant.

The MPC objective combines tracking accuracy, control effort, and steering smoothness. In the main implementation, the stage cost penalizes lateral error, heading error, steering magnitude, and steering change relative to the previous steering action. The weights are chosen to emphasize lateral tracking while discouraging abrupt steering variation. The optimization is solved with OSQP and falls back to SCS if needed. In addition to the first steering action, the controller records diagnostic quantities including solver time, objective value, tracking cost, control cost, smoothness cost, and whether the steering bound is active. These diagnostics are stored in the rollout logs for later analysis.

A simple speed-tracking heuristic produces a nominal throttle signal from the speed error, but the present work does not interpret this longitudinal heuristic. Its role is only to maintain basic forward motion so that braking policies can be studied in realistic closed-loop traffic interaction.

### 3.3 Traffic environment and scenario logging

The main braking experiments are conducted in multi-vehicle CARLA traffic environments. Background traffic is spawned from map spawn points and assigned heterogeneous behavioral profiles through the CARLA traffic manager. Three traffic profiles are defined: conservative, normal, and aggressive. These profiles differ in parameters such as speed difference, following distance, lane-change aggressiveness, and traffic-rule compliance. In the more aggressive configuration, vehicles can drive faster than the nominal flow, follow at shorter distances, and change lanes more frequently. The script uses an explicit profile mixture to create diverse interaction conditions, and the assigned traffic profile idis stored in the rollout log for each scenario.

In addition to the main multi-traffic setting, the project also contains a blockage-based scenario generator in which the ego vehicle encounters a near-stationary vehicle ahead and evaluates whether the left lane is safe for temporary borrowing. This scenario is managed through a dedicated mode manager with discrete modes such as normal lane keeping, following, borrowing the opposite lane, returning to lane, and stop-wait. Although that module is not the core focus of the braking-XAI paper, it confirms that the codebase is structured around interpretable traffic situations rather than purely random black-box rollouts.

All rollout data are stored through the MPCRecorddataclass, which includes metadata, ego kinematics, tracking and path features, traffic interaction features, lane availability, lane-gap measurements, density level, rule-context variables, control outputs, solver diagnostics, and outcome labels such as lane departure and collision flag. This logging design is important because it preserves not only the final brake value but also the contextual variables needed to explain why that brake value was generated.

### 3.4 Structured brake-state representation

The brake controller does not operate on raw images or latent end-to-end embeddings. Instead, it receives a structured state representation constructed from semantically meaningful traffic, ego-motion, and control-history variables. The representation is designed to capture four complementary aspects of the driving situation: forward hazard structure, surrounding traffic context, ego dynamic state, and short-term control memory. In particular, it includes quantities related to front-vehicle spacing, closing behavior, local traffic density, vehicle speed regulation, previous control actions, and limited lateral context. The full feature definition is summarized in Table 1.

**TABLE1:** Structured feature set used for distilled-policy training and surrogate modeling.

Feature	Category	Description	Expected role in braking
Front vehicle distance	Forward risk	Distance from ego vehicle to the nearest valid front vehicle in a lane-aligned corridor	Primary forward hazard cue
Time to collision front	Forward risk	Estimated TTC to the front vehicle based on positive closing speed	Collision imminence cue
Front vehicle rel speed	Forward risk	Ego speed minus front-vehicle speed	Closing behavior cue
Nearest vehicle distance	Traffic context	Distance to the closest nearby vehicle regardless of strict lane alignment	Local interaction context
Local vehicle count 20 m	Traffic context	Number of nearby vehicles within a 20 m radius	Short-range density cue
Local vehicle count 40 m	Traffic context	Number of nearby vehicles within a 40 m radius	Broader density cue
Speed	Ego state	Ego vehicle speed	Motion-state cue
Speed error	Ego state	Difference between target speed and current speed	Speed regulation context
Previous brake	Control memory	Brake command from the previous control step	Temporal continuity cue
Previous throttle	Control memory	Throttle command from the previous control step	Temporal control context
Previous steer	Control memory	Steering command from the previous control step	Coupled control context
Heading error	Lateral context	Heading deviation from the local reference path	Auxiliary lateral context
Lateral error	Lateral context	Lateral deviation from the reference path	Auxiliary lateral context
Distance to center line	Lateral context	Distance from ego position to lane centerline	Auxiliary lane position cue

These variables are computed directly from CARLA state and relative geometry. Front-vehicle distance is determined by scanning actors ahead of the ego vehicle within a lane-aligned corridor defined by the ego forward axis and a lateral band proportional to lane width. Relative speed is computed as ego speed minus front-vehicle speed. TTC is computed from front distance and positive closing speed, with a large sentinel value used when the relative speed is non-closing or when no valid front vehicle exists. Local density is represented by the number of nearby vehicles within fixed spatial radii, and nearest-vehicle distance captures the closest local interaction regardless of strict lane alignment. The representation also retains previous brake, throttle, and steer to preserve temporal continuity effects in learned and surrogate models.

Before training learned or surrogate models, unavailable front-distance-related variables are consistently replaced with a large sentinel value, and missing front relative speed is filled with zero. This design preserves the distinction between the absence of valid forward interaction and a finite but safe forward distance, while keeping the rollout data usable for downstream modeling.

### 3.5 Threshold-based brake policy

The first braking mechanism is a threshold-based brake policy. This controller maps a small set of manually specified safety conditions to discrete braking responses. In the implementation, if a valid front vehicle exists and TTC falls below a critical threshold, the controller issues strong braking. Slightly larger but still hazardous TTC values activate an intermediate braking level. Additional braking can also be triggered by short front distance or by sufficiently large positive closing speed under limited spacing. Finally, when the ego vehicle is already moving very slowly and the remaining front distance is not critical, the brake command is suppressed to avoid unnecessary hold braking.

This policy is fully transparent because its decision mechanism is specified through explicit conditions and fixed response levels. At the same time, it is sparse and event driven. Most ordinary states map to no braking, while specific threshold crossings activate distinct brake levels. For this reason, the threshold policy is used as the most interpretable baseline and as a conceptual contrast to the smoother risk-aware reference policy.

### 3.6 Risk-aware reference policy

The second braking mechanism is a risk-aware reference policy. Rather than issuing brake commands directly from isolated trigger conditions, this controller first converts several forward-driving cues into normalized risk components. In the implemented design, the reference policy uses four components: distance risk, TTC risk, relative-speed risk, and local-density risk. Distance risk increases as the front-vehicle distance moves into a shorter and more safety-critical range. TTC risk increases as the estimated TTC decreases into a hazardous range. Relative-speed risk is defined only for positive closing speed, and density risk increases with local traffic count. All risk components are clipped to [0*,* 1].

These components are then fused into a composite risk score through an asymmetric weighted aggregation that intentionally prioritizes forward proximity and imminent collision risk while allowing relative motion and local density to act as contextual modifiers. The composite risk score is finally mapped to a continuous brake output through piecewise intervals corresponding to low-risk, mild-risk, moderate-risk, and high-risk regimes. An additional low-speed suppression rule limits brake output when ego speed is very low and the front spacing remains noncritical. This design yields smoother and more graded brake behavior than the threshold policy while still preserving interpretable safety semantics.

Because this controller is explicit, semantically structured, and already more continuous than hard threshold logic, it serves as the *reference policy* in the remainder of the paper. The distilled brake policy is trained from rollout data generated under this reference-policy behavior.

### 3.7 Distilled brake policy

The third braking mechanism is a distilled brake policy learned from reference-policy rollouts. The training target is the brake command mpc brakegenerated by the reference policy during closed-loop simulation. The input is the same 14-dimensional structured brake state described above. Formally, the distilled policy is trained to approximate

$$u^{break}\approx f_{\theta }\left(x_{t}\right),$$ (3)

where *x*_*t*_ is the structured brake-state vector and *u* ^brake^ is the reference-policy brake output recorded in the rollout log.

The training script loads rollout CSV files indexed by town and source policy, removes samples with missing target values, excludes collision states, filters out very-low-speed samples, replaces invalid distance-related entries with a large sentinel value, fills missing front relative speed with zero, and then drops any remaining missing feature rows. A fixed train-test split with test size 0.2 and random seed 42 is used. The resulting regression model is an XGBoost regressor with 350 trees, maximum depth 5, learning rate 0.05, subsample 0.9, column subsample 0.9, squared-error objective, and *L*_2_ regularization parameter *λ* = 1.0. Performance is reported through MAE, RMSE, and *R* ^2^ on both train and test partitions.

A key technical detail is that the distilled policy is not deployed as a raw learned prediction. During online rollout, the learned prediction is first multiplied by a gain factor and clipped to [0*,* 1]. Very small predicted brake values are suppressed to zero. A fallback brake command is then computed using the reference-policy risk-score logic. If a hand-defined risky condition is active, namely, low TTC, short front distance, or sufficiently large positive closing speed within a moderate front range, the deployed brake output becomes the maximum of the amplified learned brake and the fallback brake. Otherwise, the controller uses the learned brake alone. As a result, the deployed distilled controller is neither a purely hand-crafted controller nor an unrestricted learned predictor. It is a hybrid learned safety controller whose structure still retains explicit safety fallback semantics.

This design is especially important for interpretability. Because the distilled policy is trained on structured rollout data and then deployed with an explicit fallback rule, its behavior remains analyzable in terms of forward risk features, temporal action continuity, and partial preservation of reference-policy semantics.

### 3.8 Surrogate modeling for policy explanation

To explain the realized brake behavior of each policy under deployment, the framework introduces a second modeling layer in the form of surrogate regressors. For each town and each brake policy, a separate XGBoost surrogate model is trained to predict the realized brake output from the same 14-dimensional structured feature set. The target remains mpc brake, but now it reflects the deployed policy behavior rather than only the offline reference-policy target used in distillation.

The surrogate training pipeline mirrors the data-cleaning rules used in the distilled-policy training stage. Samples with missing targets are removed, collision states are excluded, samples with speed below 0.2 m/s are filtered out, invalid front-distance, TTC, and nearest-vehicle-distance entries are replaced with a large sentinel, and missing front relative speed is filled with zero. The dataset is split with the same test fraction and random seed. The surrogate regressor uses 400 trees, maximum depth 6, learning rate 0.05, subsample 0.9, column subsample 0.9, squared-error objective, and *L*_2_ regularization *λ* = 1.0. In addition to train and test MAE, RMSE, and *R* ^2^, the script records brake-positive ratio, mean brake, and mean positive brake. A predicted-versus-ground-truth scatter plot is also generated for fidelity inspection.

These fidelity measurements are central to the interpretability argument of the paper. Since SHAP is applied to the surrogate rather than directly to the procedural controller implementation, high surrogate fidelity is used as evidence that the surrogate captures the effective brake-decision structure of the deployed policy closely enough for attribution analysis to be meaningful.

### 3.9 SHAP-based interpretability analysis

After surrogate training, SHAP TreeExplainer is applied to analyze the contribution of each brake-state variable to the predicted brake output. For each policy-town combination, the pipeline first generates a global analysis on a random sample of up to 3,000 rollout states. The script outputs both SHAP beeswarm plots and mean absolute SHAP bar plots, and it also stores ranked feature-importance tables as CSV files. In addition, it produces a local waterfall explanation for one representative state.

To avoid conclusions being dominated by the large number of zero-brake states, the same SHAP analysis is repeated on two restricted subsets. The first is a braking-only subset defined by mpc brake > 0.01. The second is a nonzero-TTC subset defined by states with finite front TTC rather than the sentinel no-interaction value. If each subset contains at least 50 valid samples, a random sample of up to 2000 states is used for SHAP analysis. These subset designs allow the paper to distinguish broad global regularities from the narrower states in which actual braking is active or meaningful forward interaction is present.

The same surrogate-plus-SHAP pipeline is applied across at least two maps, including Town10HD and Town05, and across multiple brake-policy types, including threshold, reference, and distilled policies. This makes it possible to compare whether the dominant semantic drivers of braking remain stable across deployment environments and whether the distilled policy preserves, compresses, or alters the explanation structure of the reference policy.

## 4 Experimental setup

### 4.1 Simulation platform and evaluation scope

All experiments are conducted in CARLA closed-loop simulation using the braking analysis pipeline described in Section 3. The study focuses on brake-generation behavior rather than full-stack driving performance. The primary maps considered in the current pipeline are Town10HD and Town05. In the training scripts, the distilled brake policy is trained from reference-policy rollout data in Town10HD, whereas the surrogate and SHAP analysis scripts are configured for Town05 as a representative evaluation case.

The broader empirical comparison is therefore organized around two complementary roles. Town10HD serves as the main environment for policy generation and for analyzing more frequent braking interaction under dense traffic conditions, whereas Town05 serves as a cross-town evaluation environment with different braking frequency and braking intensity characteristics. This design supports both within-town interpretation and cross-town comparison of explanation structure.

The evaluated brake policies are threshold, reference, and distilled. However, the scientific emphasis is placed on the reference and distilled policies. The threshold policy is retained mainly as an interpretable baseline that illustrates the difference between sparse trigger-based braking and continuous risk-aware braking.

The reference policy and the distilled policy form the main comparison pair for analyzing explanation-structure alignment, explanatory compression, and the extent to which a forward-risk-oriented braking structure is retained after policy distillation. In the revised experiments, this comparison is further refined through fallback-aware and quantitative analyses, so that the apparent semantic alignment between the reference and distilled policies can be interpreted more carefully across environments.

### 4.2 Traffic configuration and closed-loop rollouts

Two closely related rollout environments are used in the codebase. The general multi-traffic MPC logging script uses 100 background vehicles with a mixed traffic profile composed of 10% conservative, 50% normal, and 40% aggressive agents, together with a fixed random seed of 42. The braking-XAI rollout script uses a slightly more aggressive evaluation setting with 80 background vehicles and a traffic profile mixture of 10% conservative, 35% normal, and 55% aggressive agents, again with random seed 42. In both settings, the CARLA traffic manager is used with global leading distance 2.5 m and asynchronous traffic mode.

The ego vehicle is spawned from a fixed spawn point, and the scenario is then run in closed loop for continuous logging. In the multi-traffic script, the scenario identifier is set to multitraffic 001and the scenario type is dense mixed traffic, with a nominal target speed of 8.0 m/s. During rollout, the controller records frame index, timestamp, ego kinematics, path-tracking variables, traffic interaction features, lane-gap variables, control actions, solver diagnostics, and outcome indicators into CSV logs. These logs constitute the raw dataset for subsequent learned-policy training and surrogate explanation.

### 4.3 Lateral MPC configuration

The lateral controller used throughout the experiments is the bicycle-model MPC described in Section 3. In the multi-traffic setting, the controller uses a 0.1 s discretization step, horizon length 12, wheelbase 2.8 m, maximum steering 0.6, and maximum steering increment 0.14. The stage-cost weights are *q*_*ey*_ = 12.0, *q*_*eψ*_ = 8.0, *r*_*δ*_ = 0.6, and *r*_Δ*δ*_ = 4.0. These settings prioritize lane-tracking quality while discouraging abrupt steering variation.

The speed-tracking logic is heuristic rather than learned. If the speed error exceeds 0.5 m/s, throttle is increased proportionally up to 0.45. If the ego vehicle is substantially above the target speed, throttle is reduced and a small deceleration brake can be applied in the general MPC scripts. In the braking-XAI setting, the main brake under analysis is not this nominal speed-tracking brake but the explicit brake-policy output studied in the paper.

### 4.4 Distilled-policy training protocol

The distilled brake policy is trained from reference-policy rollout logs. In the current training script, the source policy is set to risk score, the source town is Carla Maps Town10HD, and the target variable is mpc brake. The training feature set contains the 14 structured brake-state variables defined in Table 1. Data cleaning follows a fixed procedure: samples with missing targets are removed, collision states are excluded, samples with speed below 0.2 m/s are filtered out, front-distance-related invalid markers of 1.0 are replaced with 999.0, missing front relative speed is filled with 0.0, and any remaining feature-missing rows are dropped.

The cleaned dataset is split into training and test partitions using an 80/20 split with random seed 42. The distilled model is an XGBoost regressor with 350 trees, maximum depth 5, learning rate 0.05, subsampling 0.9, column subsampling 0.9, squared-error objective, and *L*_2_ regularization parameter *λ* = 1.0. Performance is evaluated using MAE, RMSE, and *R* ^2^ on both training and test sets, and the trained model, feature definition, and metrics summary are saved to disk. The configurations of the distilled-policy model and the surrogate models are summarized in Table 2.

**TABLE 2:** Configuration of the distilled-policy model and surrogate models.

Model	Target	Feature count	Configuration
Distilled brake policy	Reference policy brake output (mpc brake)	14	XGBoost regressor with 350 trees, maximum depth 5, learning rate 0.05, subsample 0.9, column subsample 0.9, squared-error objective, *L*_2_regularization *λ*= 1.0, train/test split 80/20, random seed 42.
Surrogate model	Deployed brake output (mpc brake)	14	XGBoost regressor with 400 trees, maximum depth 6, learning rate 0.05, subsample 0.9, column subsample 0.9, squared-error objective, *L*_2_regularization *λ*= 1.0, train/test split 80/20, random seed 42.

### 4.5 Surrogate training protocol

For interpretability analysis, each evaluated brake policy is paired with a dedicated surrogate regressor trained to reproduce the deployed brake output from the same 14-dimensional structured brake-state representation used in the distillation pipeline. In the current implementation, surrogate training is performed separately for each town-policy combination so that the effective brake behavior of each deployed controller can be analyzed in its own operational context. The main evaluations reported in this paper focus on the reference and distilled brake policies in Town10HD and Town05.

The data-cleaning procedure mirrors that used in distilled-policy training. Samples with missing brake targets are removed, collision states are excluded, and low-speed states below 0.2 m/s are filtered out. Invalid front-distance, TTC, and nearest-vehicle-distance entries are replaced with a large sentinel value of 999.0, and missing front relative speed is filled with zero. After preprocessing, the dataset is split into training and test partitions using an 80/20 split with random seed 42. This consistent preprocessing pipeline ensures that surrogate fidelity comparisons across towns and policies are based on the same structured representation and filtering rules.

The surrogate model is implemented using XGBoost with a slightly larger configuration than the distilled policy model, namely, 400 trees, maximum depth 6, learning rate 0.05, subsampling 0.9, column subsampling 0.9, squared-error objective, random seed 42, and *L*_2_ regularization parameter *λ* = 1.0. In addition to MAE, RMSE, and *R* ^2^, the training script records several policy-level statistics, including brake-positive ratio, mean brake, and mean positive brake, and also generates a predicted-versus-ground-truth scatter plot for visual fidelity inspection. These outputs are later summarized in Table 3 and used in the surrogate-fidelity analysis in Section 5.

**TABLE 3:** Quantitative summary of surrogate fidelity, policy-level brake behavior, and distilled-policy fallback activation.

Town	Policy	Brake-positive ratio	Mean brake	Mean positive brake	Test RMSE	Test *R* ^2^	Fallback activation ratio
Town10HD	Reference	0.1140	0.0546	0.4794	0.0358	0.9754	–
Town10HD	Distilled	0.1509	0.0623	0.4129	0.0370	0.9616	0.7855
Town05	Reference	0.0355	0.0178	0.5011	0.0108	0.9923	–
Town05	Distilled	0.0402	0.0271	0.6729	0.0458	0.9552	0.00084

The resulting surrogate models achieve high fidelity across all reported settings. In Town10HD, the reference-policy surrogate reaches a test RMSE of 0.0358 and test *R* ^2^ of 0.9754, while the distilled-policy surrogate reaches a test RMSE of 0.0370 and test *R* ^2^ of 0.9616. In Town05, the reference-policy surrogate achieves a test RMSE of 0.0108 and test *R* ^2^ of 0.9923, whereas the distilled-policy surrogate achieves a test RMSE of 0.0458 and test *R* ^2^ of 0.9552. These values indicate that the deployed brake outputs remain highly recoverable from the structured feature space, thereby supporting the use of surrogate-based SHAP analysis as a meaningful approximation of the underlying brake-decision structure. At the same time, the consistently higher fidelity of the reference-policy surrogate suggests that the explicit reference controller is slightly more structurally regular than the deployed distilled controller.

The policy-level brake statistics reveal a more nuanced difference between environments than braking frequency alone. Town10HD exhibits more frequent braking interaction than Town05, as reflected in the higher brake-positive ratios and larger mean brake values for both policies. However, Town05 exhibits stronger mean positive brake magnitude once braking is activated, with mean positive brake values of 0.5011 *versus* 0.4794 for the reference policy and 0.6729 *versus* 0.4129 for the distilled policy. The two environments therefore differ in both braking frequency and braking intensity structure. Town10HD remains the primary analysis environment not because every braking event is stronger, but because it provides more frequent active-braking and interaction states for explanation analysis.

For the distilled controller, the updated rollout logs additionally record whether the explicit fallback mechanism is active at each control step. This makes it possible to separate the deployed hybrid behavior into fallback-active and fallback-inactive subsets for subsequent surrogate and SHAP analysis. In the current experiments, fallback activation is highly environment-dependent, occurring in 78.55% of Town10HD distilled samples but only 0.084% of Town05 distilled samples. These records are included to distinguish explanation structure arising from the learned component itself from explanation structure reinforced by the hand-coded safety fallback.

### 4.6 SHAP evaluation protocol

SHAP analysis is carried out on the trained surrogate models rather than directly on the procedural brake-policy code. In the brake-specific SHAP script, the example configuration again targets Carla Maps Town05and the learned from risk scoresurrogate model, although the same pipeline is intended to be repeated across other policy-town combinations. The analysis uses SHAP TreeExplainer and begins with a global sample of up to 3,000 rollout states drawn at random with seed 42. For this sample, the script generates a SHAP beeswarm summary plot, a SHAP bar plot based on mean absolute attribution, a CSV feature-ranking table, and a representative local waterfall explanation.

To avoid letting the large number of non-braking states dominate the explanation results, the analysis is repeated on two restricted subsets. The first subset contains only braking states satisfying mpc brake > 0.01. The second subset contains only states with finite forward interaction, operationalized as time to collision front < 999.0. If at least 50 valid samples exist in a subset, up to 2000 states are randomly sampled for SHAP analysis using the same seed. These subset settings define the protocol used later in the braking-only and nonzero-TTC result sections.

For the distilled controller, the SHAP evaluation is further extended to fallback-aware subsets defined by whether the deployed safety fallback is active. The rollout logs record a binary fallback activeindicator, which allows the deployed hybrid behavior to be separated into fallback-active and fallback-inactive samples. This fallback-aware analysis is especially important in Town10HD, where fallback activation is frequent, and provides a cleaner view of the learned component in Town05, where fallback activation is negligible. The fallback-aware protocol is introduced specifically to distinguish explanation structure arising from the learned component itself from explanation structure reinforced by the hand-coded safety fallback.

In addition to visual comparison of SHAP plots, quantitative similarity analysis is used to compare the explanation structures of the reference and distilled policies. In the current study, this is implemented through ranking-based similarity measures, including the Spearman rank correlation between mean absolute SHAP feature rankings and top-k overlap statistics. These measures provide an explicit quantitative characterization of semantic alignment, rather than relying only on side-by-side visual inspection of SHAP bar plots.

### 4.7 Experimental outputs and reporting

The experimental pipeline produces four types of outputs. First, rollout scripts generate raw CSV logs containing state, control, and traffic features. Second, distilled-policy training produces learned-model artifacts and train/test regression metrics. Third, surrogate training produces surrogate-model artifacts, fidelity metrics, and predicted-versus-true scatter plots. Fourth, SHAP analysis produces global explanation plots, subset explanation plots, local waterfall plots, and ranked mean-absolute-SHAP tables saved as CSV files. Together, these outputs support both the quantitative and qualitative analyses reported in the next section.

## 5 Results

### 5.1 Surrogate fidelity and policy-level brake behavior

Before interpreting feature attributions, it is necessary to verify that the surrogate models reproduce the deployed brake behavior with sufficiently high fidelity. Table 3 summarizes the surrogate fidelity and policy-level brake statistics for the reference and distilled policies in Town10HD and Town05. Across all evaluated settings, the surrogate regressors achieve high test *R* ^2^, indicating that the deployed brake outputs remain structurally recoverable from the 14-dimensional structured brake-state representation. This supports the use of SHAP-based attribution as a meaningful approximation of the effective brake-decision mechanism.

**FIGURE 2:**
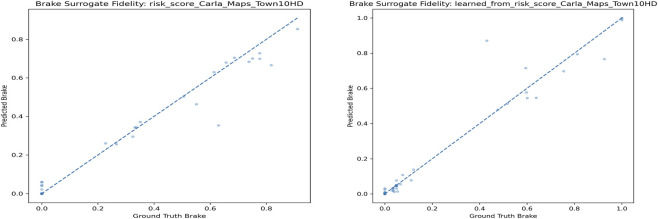
Surrogate fidelity in Town10HD for the reference policy (left) and the distilled policy (right). The reference-policy surrogate remains slightly closer to the identity line, while the distilled-policy surrogate still shows strong overall agreement with the deployed brake output.

A consistent pattern is that the surrogate models fit the reference policy more closely than the distilled policy. In Town10HD, the reference-policy surrogate reaches a test *R* ^2^ of 0.9754 with RMSE 0.0358, whereas the distilled-policy surrogate reaches a test *R* ^2^ of 0.9616 with RMSE 0.0370. As shown in Figure 2, both surrogate models remain strongly aligned with the identity line in Town10HD, although the distilled-policy surrogate exhibits slightly larger dispersion. In Town05, the same ordering remains visible, with the reference-policy surrogate achieving a test *R* ^2^ of 0.9923 and RMSE 0.0108, compared with 0.9552 and 0.0458 for the distilled policy. These results indicate that the distilled policy remains highly recoverable, but its deployed brake behavior is consistently somewhat less directly recoverable than that of the explicit reference policy. This trend is consistent with the interpretation that distillation yields a more compact or behaviorally compressed deployed control structure.

**FIGURE 3:**
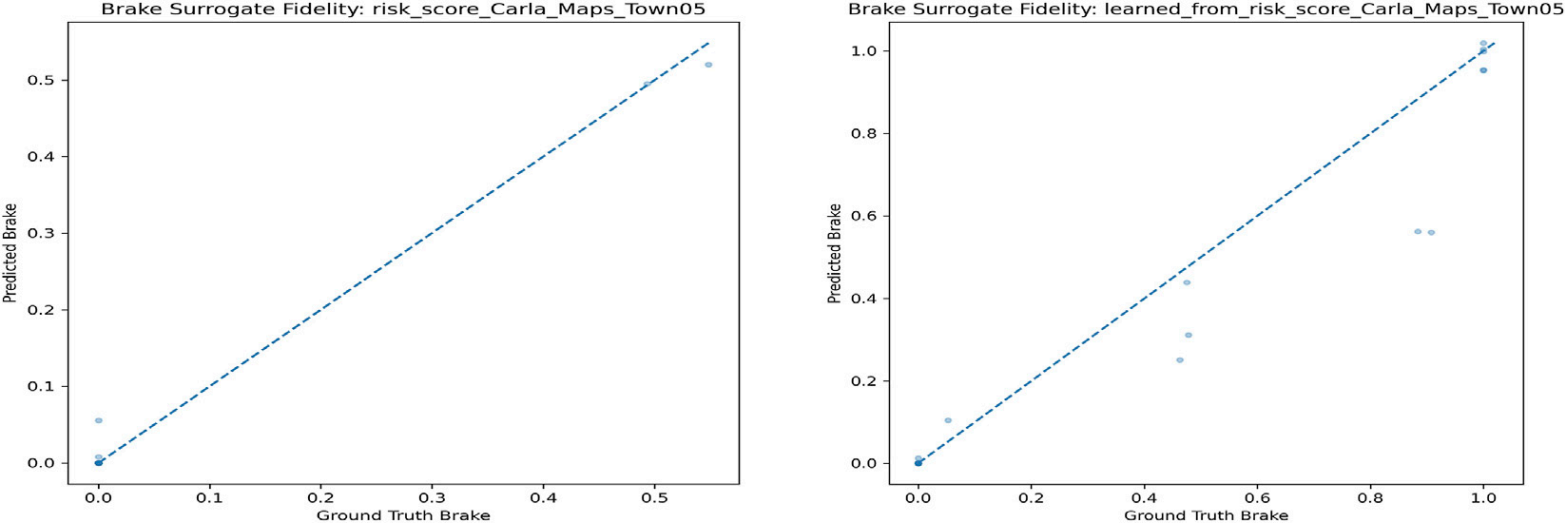
Surrogate fidelity in Town05 for the reference policy (left) and the distilled policy (right). The Town05 reference policy is especially close to the identity line, whereas the distilled policy remains highly correlated but exhibits slightly larger dispersion.

The policy-level brake statistics further reveal a more nuanced difference across towns than braking frequency alone. Town10HD exhibits more frequent braking interaction than Town05, as reflected in the higher brake-positive ratios and larger mean brake values for both policies. However, Town05 exhibits stronger mean positive brake magnitude once braking is activated, with mean positive brake values of 0.5011 *versus* 0.4794 for the reference policy and 0.6729 *versus* 0.4129 for the distilled policy. The two environments therefore differ in both braking frequency and braking intensity structure. Town10HD remains the primary analysis environment not because every braking event is stronger, but because it provides more frequent active-braking and interaction states for explanation analysis. As shown in Figure 3, the same fidelity pattern remains visible in Town05, where the reference-policy surrogate is especially close to the identity line and the distilled-policy surrogate remains highly correlated but somewhat more dispersed.

The comparison between the reference and distilled policies also reveals a consistent behavioral difference. In both towns, the distilled policy produces a slightly higher brake-positive ratio than the reference policy, indicating that it enters the braking regime somewhat more often. In Town05, the mean positive brake is also larger for the distilled policy than for the reference policy, whereas in Town10HD the mean positive brake is lower for the distilled policy despite its higher activation frequency. This suggests that the distilled policy does not simply imitate the reference policy through uniformly stronger braking. Instead, it appears to reorganize the brake behavior by changing both the frequency and intensity structure of positive brake outputs.

An additional important result comes from the direct distillation stage. On held-out Town10HD data generated from the reference policy, the distilled brake policy achieves a test *R* ^2^ of 0.9783 with RMSE 0.0336. This shows that the mapping from the structured brake-state representation to the reference brake output is highly learnable in the selected feature space.

At the same time, the interpretation of the distilled-policy results requires additional care because the deployed controller is hybrid rather than purely learned. The updated rollout logs show that fallback activation is highly town-dependent, occurring in 78.55% of Town10HD distilled samples but only 0.084% of Town05 distilled samples. This implies that the full-policy explanations in Town10HD should be interpreted as explanations of a hybrid deployed controller, whereas Town05 provides a much cleaner view of the learned component itself. Accordingly, the apparent alignment between the reference and distilled policies in Town10HD cannot be attributed to the learned model alone without fallback-aware analysis.

Figure-based fidelity inspection is consistent with these quantitative results. The surrogate-fidelity scatter plots show that the Town05 reference policy is especially close to the diagonal, while the Town10HD distilled policy exhibits slightly larger dispersion around the identity line, despite remaining strongly correlated overall. This visual difference matches the numerical fidelity gap observed in Table 3 and reinforces the conclusion that the reference policy is somewhat more explicit and structurally regular than the distilled policy under deployment.

### 5.2 Global explanation structure across policies and towns

**FIGURE 4:**
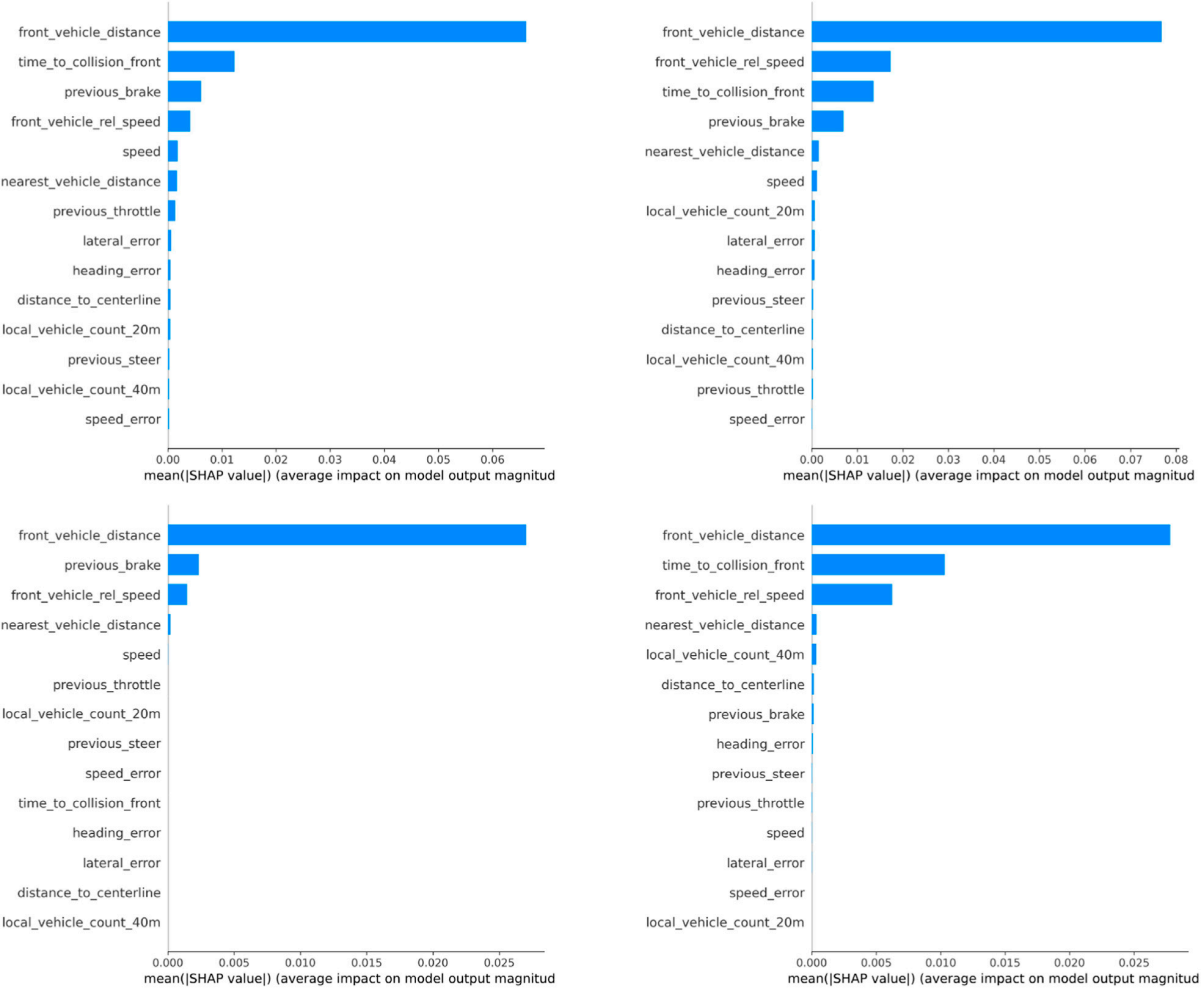
Global SHAP bar plots for the reference and distilled brake policies across Town10HD and Town05. Across all settings, front vehicle distanceremains the dominant brake determinant. The reference policy exhibits a more explicit multi-factor hierarchy centered on distance, TTC, and relative speed, whereas the distilled policy shows a more compact structure with a stronger role for previous brake, especially in Town05.

The global SHAP analysis reveals a stable explanatory structure across towns and policies. In all evaluated settings, braking is dominated by a compact set of forward-interaction variables rather than by lateral tracking variables. Most notably, front vehicle distanceis the strongest brake determinant throughout the study. This pattern appears in both Town05 and Town10HD and remains visible for both the reference and distilled policies. As shown in Figure 4, the global importance ordering is highly stable across towns, with front vehicle distanceconsistently ranked first and forward-interaction variables dominating the explanation hierarchy. The result suggests that brake generation in the present framework is governed primarily by forward hazard structure rather than by lane-tracking deviation or other auxiliary vehicle-state variables.

For the reference policy, the global feature hierarchy is especially clear. In Town05, the dominant explanatory variables are front vehicle distance, time to collision front, and front vehicle rel speed, with only small residual contributions from variables such as nearest vehicle distance, local density terms, and weak lateral-context variables. The Town10HD reference policy shows the same overall ordering, with front distance remaining the strongest contributor and TTC and relative speed continuing to play major roles. This consistency indicates that the reference policy preserves an explicit multi-factor risk structure across environments, centered on forward proximity, collision imminence, and closing behavior.

The interpretation of the distilled-policy results requires additional care because the deployed distilled controller is hybrid rather than purely learned. The fallback-aware rollout statistics show that fallback activation is highly environment-dependent, occurring in 78.55% of Town10HD distilled samples but only 0.084% of Town05 distilled samples. This means that the full-policy explanation in Town10HD should be interpreted as the explanation of a hybrid deployed controller, whereas Town05 provides a much cleaner approximation of the learned component itself.

The distilled policy therefore should not be described as preserving exactly the same explanation structure as the reference policy in all settings. A more precise conclusion is that the deployed distilled controller remains forward-risk-oriented, but the degree of apparent alignment with the reference policy depends on the environment and on the contribution of the fallback mechanism. In Town05, where fallback activation is negligible, the global SHAP ranking still places front vehicle distancefirst, but previous brakerises sharply in importance, while the role of time to collision frontbecomes less explicit than in the reference policy. In Town10HD, the same broad forward-risk orientation remains visible, but the full-policy explanation structure is substantially reinforced by fallback activation and therefore cannot be attributed to the learned component alone.

A second consistent observation is the weakness of lateral variables in the braking explanation hierarchy. Across towns and policies, variables such as lateral error, heading error, and distance to centerlineremain much smaller in SHAP magnitude than the principal forward-risk terms. This is important because it confirms that the brake controller is not primarily responding to lane-tracking deviation. Instead, braking is activated mainly by forward-traffic structure, while lateral quantities serve only as weak contextual modifiers.

**FIGURE 5:**
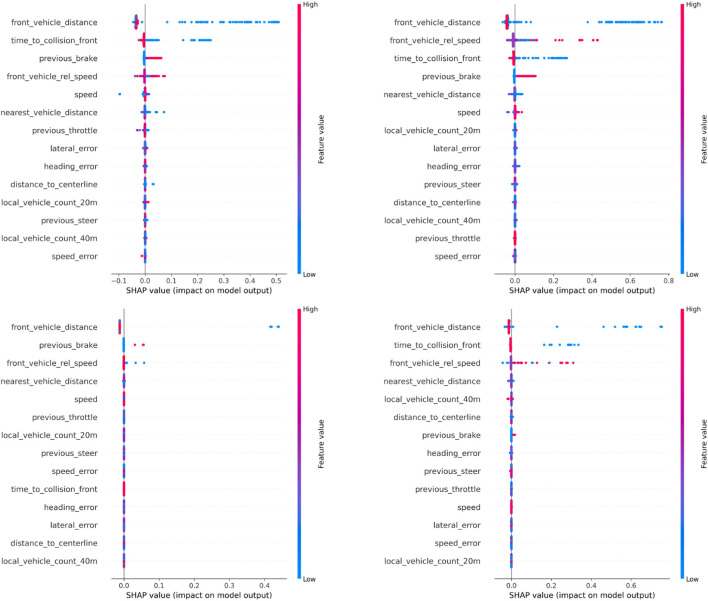
Global SHAP summary plots for the reference and distilled brake policies across Town10HD and Town05. The summary plots show that smaller front-vehicle distance, shorter TTC, and larger positive relative speed generally push the brake prediction upward. In the distilled policy, previous_brakecontributes more strongly, indicating greater temporal continuity in the deployed brake behavior.

Figure 5 further clarifies the directionality of these effects. In both towns, smaller front_vehicle_ distance, shorter time_to_collision_front, and larger positive front_vehicle_rel_speedgenerally push the brake prediction upward. At the same time, the distilled policy shows a relatively stronger contribution from previous_brake, especially in Town05, indicating that temporal continuity plays a more visible role in the deployed learned controller. The summary plots therefore complement the global ranking results by showing not only which variables matter most, but also how their values shape brake output in different environments.

**FIGURE 6:**
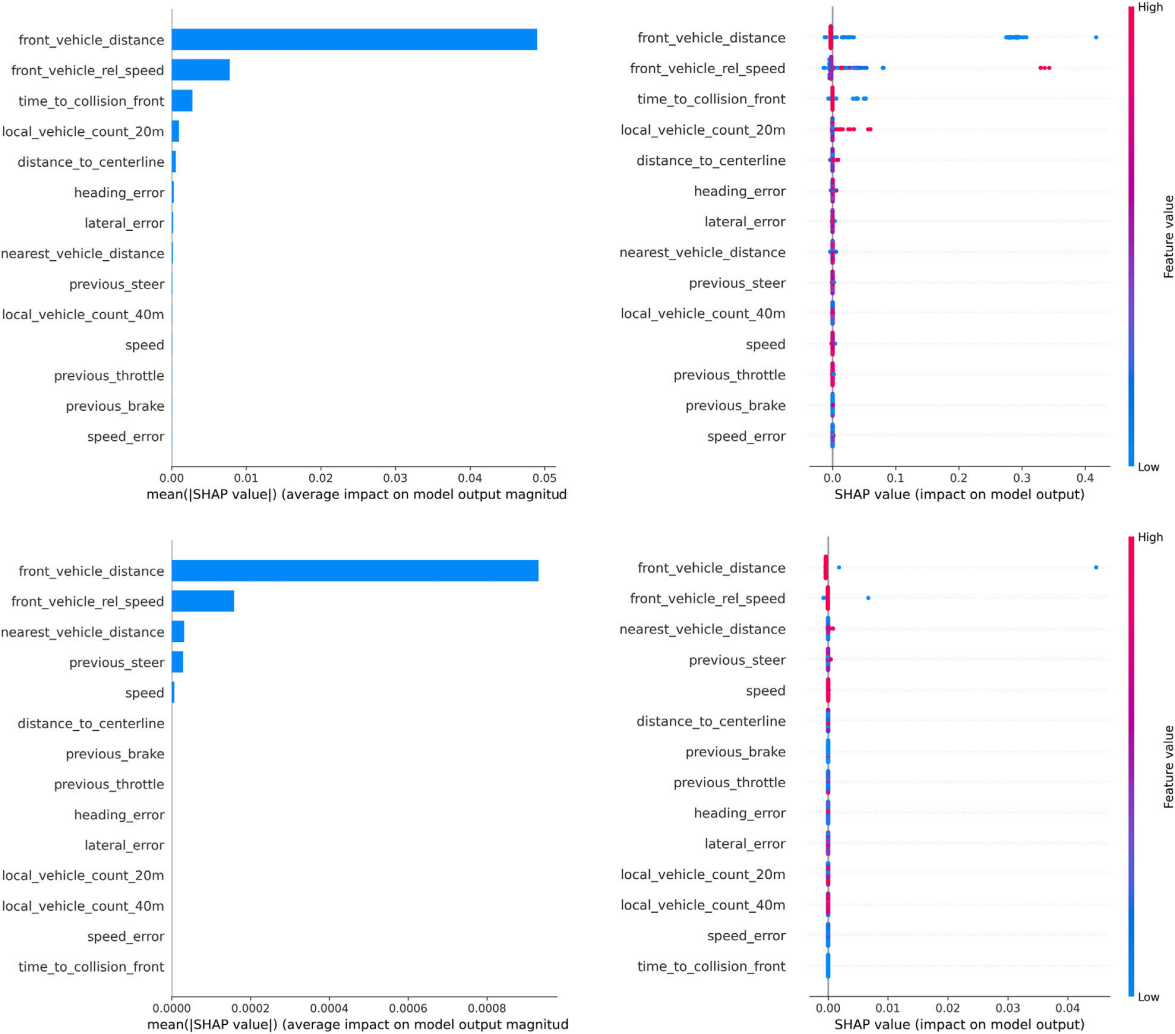
Fallback-aware SHAP analysis of the distilled controller in Town10HD and Town05. The fallback-inactive results provide a cleaner view of the learned component by reducing the influence of the deployed safety fallback. In Town10HD, the fallback-inactive explanation remains dominated by front vehicle distance, front vehicle rel speed, and time to collision front. In Town05, where fallback activation is negligible, the learned controller remains primarily governed by front vehicle distanceand front vehicle rel speed, while the role of time to collision frontbecomes much weaker.

To distinguish explanation structure arising from the learned component itself from structure reinforced by the deployed safety fallback, the distilled controller is further analyzed on fallback-inactive samples. As shown in Figure 6, the fallback-inactive explanation remains forward-risk-oriented in both towns, but the relative importance of TTC is much weaker in Town05 than in Town10HD. In Town10HD, the fallback-inactive explanation remains dominated by front vehicle distance, front vehicle rel speed, and time to collision front. In Town05, where fallback activation is negligible, the learned controller remains primarily governed by front vehicle distanceand front vehicle rel speed, while the role of time to collision frontbecomes much weaker.

To complement the visual comparison in Figure 6 and Table 4 reports quantitative similarity metrics between the reference and distilled policies based on global mean-absolute-SHAP rankings.

**TABLE 4:** Quantitative similarity between the reference and distilled policies based on global mean-absolute- SHAP rankings.

Town	Spearman ρ	Top-5 overlap	Top-10 overlap
Town10HD	0.8549	0.8	0.8
Town05	0.2756	0.6	0.7

Quantitative similarity analysis further shows that apparent semantic alignment is not uniform across towns. As summarized in Table 4, the reference and distilled policies exhibit strong global alignment in Town10HD (*ρ* = 0.8549, top-5 overlap = 0.8, top-10 overlap = 0.8), but much weaker alignment in Town05 (*ρ* = 0.2756, top-5 overlap = 0.6, top-10 overlap = 0.7). These results support a qualified semantic-preservation claim: the distilled controller retains a forward-risk-oriented explanation structure, but exact alignment with the reference policy is environment-dependent rather than uniformly strong.

The cross-town comparison also provides useful insight into environmental dependence. The explanatory structure is more information-rich in Town10HD, where braking events are more frequent and the interaction pattern is denser. Under these richer conditions, the distilled policy continues to rely strongly on front_vehicle_distance, front_vehicle_rel_speed, and time_to_collision_front, with previous_brakereinforcing rather than replacing the forward-risk hierarchy. In the sparser Town05 environment, by contrast, previous_brakebecomes more dominant relative to TTC, suggesting that temporal continuity takes a larger role when forward-interaction cues are less persistently informative. This difference does not undermine the global conclusion. On the contrary, it shows that the distilled policy adapts its explanatory balance while preserving the same dominant forward-risk foundation.

Taken together, these results support two more qualified claims. First, the broad explanation structure of braking remains forward-risk-oriented across towns, with front distance, TTC, and positive closing behavior playing central roles. Second, the distilled controller does not behave like an arbitrary black box, but the degree of semantic alignment with the reference policy is not uniform across environments. In Town10HD, the apparent alignment is strengthened by the fallback mechanism, whereas in Town05 the explanation structure provides a cleaner view of the learned component and reveals a less direct correspondence with the reference ranking.

### 5.3 Subset analysis on active braking and forward interaction

The global SHAP analysis in Section 5.2 shows that brake generation is dominated by forward-risk variables across towns and policies. However, a potential concern is that these global results may be influenced by the large number of zero-brake states present in closed-loop rollout data. To address this issue, the analysis is repeated on two more targeted subsets: a *braking-only subset* containing states with positive brake output, and a *nonzero-TTC subset* containing states with valid forward interaction rather than the no-interaction sentinel condition. These subset analyses provide a more focused view of the brake mechanism under active braking and meaningful front-vehicle interaction.

The braking-only subset is particularly important because it tests whether the global feature hierarchy is merely a byproduct of the strong imbalance between non-braking and braking states. If the global explanation pattern were mainly driven by the abundance of zero-brake samples, then restricting the analysis to active braking states would be expected to alter the hierarchy substantially. Instead, the braking-only results in Town10HD show that the core explanation structure remains highly stable for both the reference and distilled policies.

**FIGURE 7:**
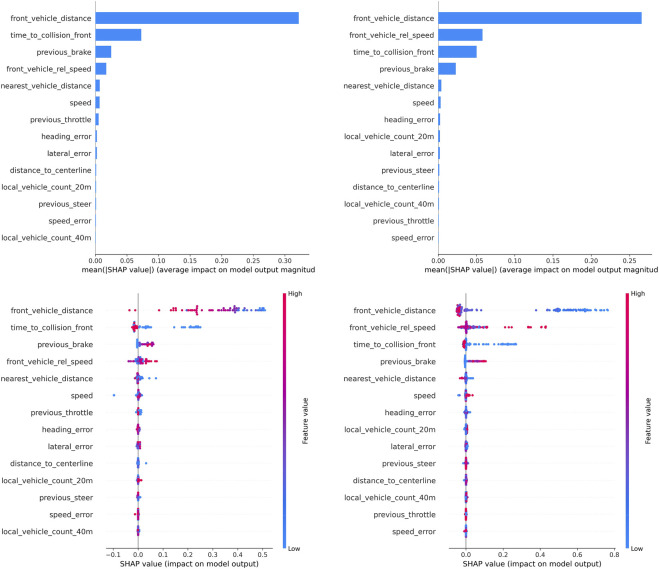
SHAP analysis on the braking-only subset in Town10HD for the reference policy (left column) and the distilled policy (right column). Even when restricted to active braking states, the dominant explanation hierarchy remains centered on front vehicle distance, time to collision front, and front vehicle rel speed. In the distilled policy, previous brakeremains a visible contributor, indicating stronger temporal continuity under active braking.

For the reference policy, the dominant features in the braking-only subset remain front vehicle distance, time to collision front, and front vehicle rel speed, indicating that once braking is actually engaged, the controller still relies on an explicit forward-risk structure centered on distance, collision imminence, and closing behavior. The distilled policy shows the same forward-risk foundation, with front vehicle distance, front vehicle rel speed, and time to collision frontremaining the leading contributors. At the same time, previous brakeremains visibly important in the distilled policy, showing that temporal continuity continues to shape brake output even when the analysis is restricted to active braking states. As shown in Figure 7, the braking-only subset preserves the same forward-risk hierarchy observed in the full dataset, indicating that the global explanation pattern is not driven by the large number of zero-brake states.

The nonzero-TTC subset provides an even more targeted test of whether TTC-related reasoning is truly meaningful. In this subset, all states correspond to situations with valid finite forward interaction, rather than cases where no front vehicle exists and TTC is effectively absent. This restriction removes the large no-interaction group and focuses the analysis on states in which collision imminence is actually well defined.

Under this restriction, the reference policy again exhibits a clear distance–TTC–relative-speed hierarchy, which supports the interpretation that TTC is not merely a weak correlational byproduct but an active component of the braking logic when forward interaction is present. The distilled policy likewise preserves a strong dependence on front distance, TTC, and relative speed, while still retaining a secondary contribution from previous brake. This indicates that the temporal continuity term does not replace collision-imminence reasoning. Instead, it coexists with the core forward-risk structure.

**FIGURE 8:**
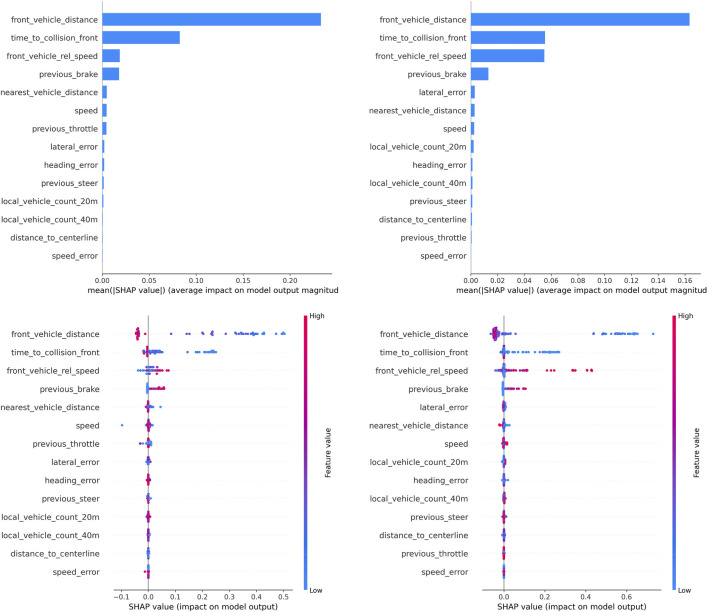
SHAP analysis on the nonzero-TTC subset in Town10HD for the reference policy (left column) and the distilled policy (right column). When attention is restricted to states with valid forward interaction, the reference policy preserves a clear distance–TTC–relative-speed structure, and the distilled policy retains the same forward-risk basis while also showing a continuity-related contribution from previous brake.

Figure 8 further shows that when attention is restricted to states with valid forward interaction, TTC remains an active explanatory variable rather than a weak byproduct of the full-state distribution.

For the distilled controller, these subset results must be interpreted together with the fallback-aware analysis reported in the revised experiments. In Town10HD, the full-policy subset explanations still reflect a hybrid deployed controller because fallback activation is frequent. However, additional fallback-inactive analysis shows that the learned component itself remains forward-risk-oriented: when fallback is inactive, the explanation structure is still dominated by front vehicle distance, front vehicle rel speed, and, in Town10HD, time to collision front. At the same time, the contribution of previous brakebecomes noticeably weaker in the fallback-inactive analysis than in the original full-policy explanation. This indicates that part of the continuity signature observed in the deployed distilled controller is amplified by hybrid deployment rather than entirely learned by the model itself.

The Town05 fallback-aware results are especially informative because fallback activation is negligible there. In this near-pure learned setting, the distilled controller still exhibits a clear forward-risk-oriented explanation structure, dominated primarily by front vehicle distanceand front vehicle rel speed. However, the role of time to collision frontbecomes much weaker than in the reference controller. This shows that the learned component does not simply inherit the exact same explanation hierarchy as the reference policy. Rather, it preserves a broader forward-risk logic while changing the relative weight of individual explanatory variables across environments.

The subset comparison therefore helps clarify the relationship between the reference and distilled policies. In the original full global analysis, the Town05 distilled policy appeared to shift more strongly toward previous brake, raising the possibility that temporal continuity might dominate the learned representation. The Town10HD subset results already showed that this interpretation would be incomplete. The additional fallback-aware results make this point more precise: the learned component of the distilled controller does not simply memorize brake persistence, but combines a retained forward-risk mechanism with environment-dependent differences in explanatory balance.

A further benefit of the subset and fallback-aware analyses is that they sharpen the interpretation of the distilled controller as a compressed but not semantically arbitrary approximation. In the reference policy, the active-braking subsets preserve the explicitly engineered risk hierarchy almost exactly. In the distilled controller, the same subsets preserve the broader forward-risk orientation, but not necessarily the exact same ranking or relative emphasis of TTC, relative speed, and temporal continuity. This supports a qualified preservation claim rather than a claim of exact semantic equivalence.

Overall, the subset analyses strengthen two conclusions. First, the core brake semantics identified in the global SHAP results remain valid when attention is restricted to the most relevant states, so the explanation hierarchy is not simply an artifact of many zero-brake or no-interaction samples. Second, the distilled controller remains forward-risk-oriented under active braking and valid forward interaction, but the degree of apparent alignment with the reference policy must be interpreted together with fallback activation and environmental context.

### 5.4 Local explanation examples

While the global and subset analyses establish the overall feature hierarchy of braking, local explanations are useful for showing how this hierarchy is instantiated in individual states. In particular, local waterfall plots make it possible to examine whether the predicted brake output in a specific sample is driven by the same semantic factors identified in the global SHAP results. In this section, representative fallback-aware waterfall explanations are used to illustrate both low-risk and positive-braking cases for the distilled controller.

We first consider low-risk or no-interaction states, in which the predicted brake output is close to zero. In such states, the fallback-inactive local explanations are fully consistent with the global explanation hierarchy. Large or sentinel-valued front vehicle distance, effectively absent forward collision imminence, and weak closing behavior all push the brake prediction downward toward zero. These conditions indicate that no meaningful front-vehicle hazard is currently present. The corresponding waterfall plots therefore provide a local confirmation that the learned component itself suppresses braking when forward proximity, collision imminence, and closing behavior are all weak or absent.

**FIGURE 9:**
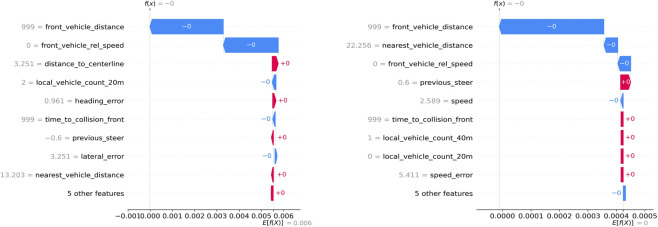
Fallback-inactive local SHAP waterfall explanations for representative low-risk states in the distilled controller. In both examples, the absence of meaningful forward hazard, reflected by large or sentinel-valued front vehicle distanceand time to collision front, together with weak closing behavior, suppresses the predicted brake output toward zero. These cases provide a cleaner view of the learned component because the explicit safety fallback is inactive.

This fallback-aware low-risk interpretation is illustrated in Figure 9. In both Town10HD and Town05, the distilled controller assigns near-zero brake under effectively non-hazardous forward conditions even when the explicit safety fallback is inactive. This is important because it shows that the learned component itself, rather than only the hybrid deployment rule, remains semantically consistent in non-braking states.

We next consider positive-braking states, where the local explanation is expected to reflect genuine forward hazard structure. In these cases, the waterfall plots show that the predicted brake output is jointly increased by short or moderate front spacing, shorter TTC, and positive closing speed. These examples are especially important because they demonstrate that the distilled controller does not merely rely on persistence or fallback-induced alignment. Instead, it still responds strongly to forward-risk variables that are physically meaningful for braking.

**FIGURE 10:**
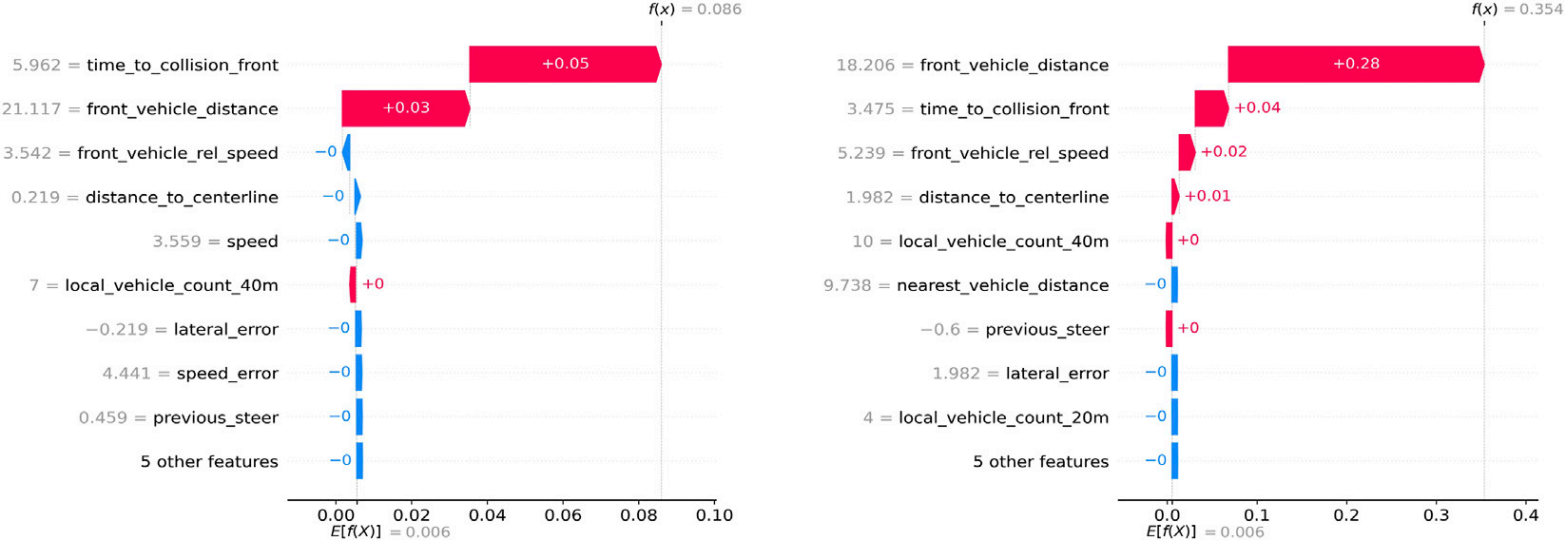
Fallback-aware local SHAP waterfall explanations for two positive-braking states of the distilled controller in Town10HD. In both examples, braking is primarily driven by forward-risk variables, especially front vehicle distance, time to collision front, and front vehicle rel speed. The stronger-braking example on the left shows a larger cumulative positive contribution from these forward-interaction variables, while the milder-braking example on the right reflects the same semantic structure at a lower brake level.

Figure 10 complements the near-zero-brake examples in Figure 9 by showing two positive-braking states from Town10HD. In both cases, the predicted brake output is increased mainly by forward-risk variables rather than by lateral or tracking-related variables. The strongest contribution comes from front vehicle distance, while time to collision frontand front vehicle rel speedprovide additional positive support. This pattern indicates that even when the explicit safety fallback is not the primary source of the explanation, the distilled controller still produces braking decisions through an interpretable forward-risk structure. The left example corresponds to a stronger braking response, whereas the right example shows a milder but semantically consistent braking decision.

These local examples strengthen the interpretation developed in the previous subsections. At the sample level, the learned component of the distilled controller remains responsive to semantically meaningful forward-risk cues rather than behaving like an arbitrary black-box mapping. At the same time, the fallback-aware results reinforce a more qualified conclusion than the original manuscript. The local explanations do not show exact semantic equivalence with the reference policy in every environment. Instead, they show that the distilled controller retains a forward-risk-oriented explanation structure whose apparent alignment with the reference policy depends on the operating environment and on the contribution of the deployed safety fallback.

Taken together, the local waterfall plots provide an intuitive bridge between global explanation structure and individual braking events. They show that the same small set of forward-risk variables drives both the average and the instance-level behavior of the controller, thereby increasing confidence that the reported explanation hierarchy reflects the genuine deployed brake mechanism rather than only an aggregate statistical trend.

## 6 Discussion

The results of this study support a practical and more nuanced view of interpretability in learning-based control. In safety-critical driving, the relevant question is often not whether a learned controller is perfectly transparent in a mechanistic sense, but whether its deployed behavior can still be examined, compared, and understood through semantically meaningful variables. Under the present framework, the answer appears to be yes. By defining brake control on structured traffic-state features, anchoring the learned controller to a semantically explicit reference policy, and analyzing deployed behavior through high-fidelity surrogate models and SHAP-based attribution, the resulting brake policy remains sufficiently interpretable for meaningful scientific analysis.

A central implication of the results is that braking in the current closed-loop setting is not governed by an arbitrarily diffuse black-box representation. Instead, both the reference and distilled policies are dominated by a compact forward-risk structure centered on front-vehicle distance, relative speed, and time to collision. This observation is important because it shows that the learned controller is still reacting to the physically relevant quantities one would expect from a safety-oriented brake mechanism. In other words, the use of a learned model does not automatically imply that the behavioral basis of control becomes untraceable. At least in the present structured setting, the dominant semantics of braking remain recoverable after learning.

At the same time, the revised results suggest that this interpretability claim should be stated more carefully for the distilled controller. The deployed distilled policy is hybrid rather than purely learned, because it includes an explicit fallback mechanism under predefined risky conditions. The additional fallback-aware analysis shows that this distinction is especially important in Town10HD, where fallback activation is frequent, whereas Town05 provides a much cleaner view of the learned component itself. Accordingly, the explanation structure observed for the full distilled controller in Town10HD should be interpreted as the behavior of a hybrid deployed controller rather than as the learned model alone.

The comparison between the reference and distilled policies provides a more specific insight. The reference policy is easier to understand directly because its design explicitly encodes forward-risk logic through engineered risk components and a graded brake mapping. The distilled policy, by contrast, is learned from rollout data and therefore no longer exposes its decision mechanism in analytic form.

Nevertheless, the revised explanation results show that the learned component of the distilled controller still retains a forward-risk-oriented braking logic. In fallback-inactive Town10HD samples, the explanation structure remains dominated by front vehicle distance, front vehicle rel speed, and time to collision front, while in Town05, where fallback activation is negligible, the distilled controller remains primarily governed by front distance and relative speed. These findings suggest that the learned controller does not merely inherit the reference policy through the hard-coded fallback rule. At the same time, they also show that exact semantic equivalence should not be claimed: the broader forward-risk orientation is preserved, but the relative emphasis of individual features changes across environments.

One of the most informative findings in this regard is the stronger contribution of previous brakein the distilled policy. This effect appears in the global analysis, remains visible in braking-only and nonzero- TTC subsets, and is also reflected in the local waterfall explanations. The role of previous brakeis important because it indicates that the learned controller is not merely mapping instantaneous forward-risk features to brake in a static way. It is also encoding temporal continuity. From a control perspective, this is plausible: once braking has begun, maintaining continuity in the brake signal can help avoid abrupt oscillations and produce smoother deployed behavior. From an interpretability perspective, however, this temporal continuity also shows that the distilled controller is not a term-by-term replica of the reference policy. It is a compact behavioral approximation that blends forward-risk reasoning with action persistence.

The fallback-aware subset analysis refines this interpretation further. In the original full-policy explanation, especially in Town10HD, the continuity signature of previous brakeis stronger because the deployed controller combines learned behavior with safety fallback. When fallback-inactive samples are analyzed separately, the contribution of previous brakebecomes weaker and the explanation structure shifts more clearly toward immediate forward-risk cues. This suggests that part of the continuity signature observed in the full deployed controller is amplified by hybrid deployment rather than entirely learned by the distilled model itself.

Town10HD and Town05 should not be interpreted as differing only in an undirected notion of “richer” or “weaker” braking. Instead, the two environments differ along at least two dimensions. Town10HD exhibits more frequent braking interaction, as reflected in the higher brake-positive ratios and larger mean brake values. Town05, however, exhibits stronger mean positive brake magnitude once braking is activated. The two towns therefore differ in both braking frequency and braking intensity structure. Under these different conditions, the explanation balance of the distilled controller also changes: Town10HD preserves a stronger multi-factor forward-risk pattern, while Town05 shows a more selective dependence on front distance and relative speed, with a weaker role for TTC.

Another important implication concerns the role of subset analysis. One possible criticism of global explanation results in closed-loop driving is that the abundance of non-braking states may dominate the interpretation and obscure the mechanism of active control. The present results help address that concern. When the analysis is restricted to braking-only states or to states with valid finite TTC, the same core structure remains visible. In particular, front distance, TTC, and relative speed remain dominant for both policies, while previous brakeretains a visible role in the distilled controller. This consistency strengthens the claim that the explanation hierarchy reflects the actual mechanism of braking rather than a statistical artifact induced by many idle states.

The revised results also show that subset analysis alone is not sufficient for the distilled controller. Braking-only and nonzero-TTC subsets confirm that the broad forward-risk orientation is not simply an artifact of zero-brake dominance, but fallback-aware analysis is still needed to determine how much of the observed structure belongs to the learned component and how much belongs to the hybrid deployment rule. In this sense, the present work highlights that interpretability claims for learned safety controllers should be made at multiple levels: full deployed behavior, fallback-aware deployed behavior, and learned-component behavior.

The local waterfall explanations further reinforce this point. At the instance level, low-risk states are consistently characterized by large front distance, effectively absent collision imminence, and weak closing behavior, all of which suppress brake output. Active-braking states, by contrast, are driven by short front spacing, shorter TTC, positive closing speed, and, in the distilled controller, continuity from previous brake. These sample-level results match the global and subset findings closely. This agreement between global and local explanation is an important strength of the present framework, because it suggests that the identified feature hierarchy operates not only as an average trend but also at the level of individual control decisions.

At the same time, the revised quantitative similarity analysis shows that semantic alignment should be treated as a measurable and qualified property rather than a purely visual impression. Using Spearman rank correlation and top-k overlap of mean absolute SHAP rankings, the reference and distilled policies exhibit strong global alignment in Town10HD but substantially weaker alignment in Town05. This means that the statement that the distilled controller “preserves the core safety semantics” of the reference policy is too strong if interpreted as exact or town-invariant semantic equivalence. A more defensible conclusion is that the distilled controller retains a forward-risk-oriented explanation structure, while the degree of apparent semantic alignment remains environment-dependent.

More broadly, the present study highlights the value of analyzing a narrowly defined safety-critical control channel rather than trying to explain an entire autonomous-driving stack at once. Full-stack autonomous driving involves perception, prediction, planning, and control, and explanations at that scale often become diffuse or ambiguous. By focusing specifically on brake generation, the present work is able to formulate a clearer interpretability question: what variables govern the decision to brake, and how does that structure change after distillation? This narrower framing makes it possible to derive more precise and defensible conclusions. In particular, it becomes possible to separate the semantics of forward-risk braking from unrelated components such as lateral path-tracking dynamics.

At the same time, the present findings should be interpreted within the scope of the current framework. The distilled policy is defined on structured features rather than raw sensory input, and the reference policy itself is semantically engineered. This means that the study does not claim that arbitrary end-to-end learned driving policies will exhibit the same degree of interpretability. Rather, the results show that when a learned controller is grounded in structured safety-relevant features and tied to a meaningful reference policy, its deployed behavior can remain interpretable enough for detailed analysis. This is an important but bounded claim.

A related limitation is that the explanation pipeline is surrogate based. SHAP is applied not directly to the procedural controller logic, but to high-fidelity XGBoost surrogates trained on deployed rollout behavior. The high surrogate fidelity observed in this study provides strong support for using these surrogates as explanation vehicles, but it does not turn the resulting attributions into exact mechanistic proofs of the controller internals. The explanations should therefore be interpreted as faithful behavioral approximations rather than literal decompositions of the original policy implementation.

Finally, the present work opens several promising directions for future research. One direction is to extend the analysis to more maps, seeds, and traffic regimes in order to test how stable the explanation hierarchy remains under broader environmental variation. A second is to study the temporal dimension more explicitly, for example, through sequence-aware models or direct analysis of brake hysteresis and action persistence. A third is to expand the framework beyond braking and compare the interpretability of longitudinal and lateral control channels under a unified analysis pipeline. A fourth is to investigate whether similar reference-to-distilled policy structures can be used for other safety-critical robotic control tasks where learning is desirable but interpretability remains a barrier to deployment.

In summary, the discussion suggests that the value of the present framework lies not in claiming exact semantic equivalence between the reference and distilled controllers, but in showing that learning-based brake control can remain substantially more interpretable when the learning problem is carefully structured and when the deployed hybrid behavior is analyzed with fallback-aware and quantitative tools. The distilled controller studied here remains a learned policy, yet its deployed behavior continues to reflect a compact and physically meaningful forward-risk logic. This provides a practical middle ground between rigid hand-crafted control and fully opaque black-box policies, while also illustrating the importance of carefully distinguishing learned behavior from safety-rule reinforcement in hybrid autonomous-control systems.

## 7 Conclusion

This paper presented an explainable analysis framework for brake generation in CARLA closed-loop driving simulation. Rather than attempting to explain a full autonomous-driving stack, the study focused on a narrower but safety-critical control channel: longitudinal braking. Within a decoupled control architecture, a bicycle-model MPC controller provided lateral tracking, while brake generation was governed by a threshold-based policy, a risk-aware reference policy, or a distilled brake policy learned from reference-policy rollouts. This formulation made it possible to study braking as a structured interpretability problem under realistic traffic interaction and deployment conditions.

The results showed that braking in the present framework is consistently dominated by a compact set of forward-risk variables. Across towns, policies, and analysis subsets, front vehicle distanceemerged as the strongest brake determinant, while front vehicle rel speedand time to collision frontalso played major roles. By contrast, lateral variables such as lateral error, heading error, and distance to centerlinecontributed only weakly. This indicates that brake generation is governed primarily by forward hazard structure rather than by lane-tracking deviation.

A second major conclusion is that the distilled controller retains a forward-risk-oriented explanation structure, but the degree of apparent semantic alignment with the reference policy is environment-dependent. Surrogate-fidelity results showed that both the reference and distilled controllers remain highly recoverable from the structured brake-state representation, although the reference policy is consistently somewhat easier to recover than the distilled policy. Global and subset SHAP analyses further showed that the distilled controller remains centered on forward-risk variables, while the relative role of temporal continuity and TTC varies across environments and subsets. These results support a qualified semantic-preservation claim rather than a claim of exact semantic equivalence.

The fallback-aware analysis provides an important refinement of this conclusion. The deployed distilled controller is hybrid rather than purely learned, because it includes an explicit safety fallback under predefined risky conditions. Fallback activation is highly environment-dependent, occurring frequently in Town10HD but only rarely in Town05. As a result, the full-policy explanations in Town10HD should be interpreted as the behavior of a hybrid deployed controller, whereas Town05 provides a much cleaner view of the learned component itself. At the same time, the fallback-inactive analyses show that the learned component still retains a semantically meaningful forward-risk structure, rather than depending entirely on the hand-coded fallback rule.

The cross-town comparison provided an additional insight into environmental dependence. Town10HD exhibited more frequent braking interaction, as reflected in the higher brake-positive ratios and larger mean brake values, whereas Town05 exhibited stronger mean positive brake magnitude once braking was activated. The two environments therefore differ in both braking frequency and braking intensity structure. Under these different conditions, the explanation balance of the distilled controller also changes: Town10HD retains a stronger multi-factor forward-risk pattern, while Town05 shows a more selective dependence on front distance and relative speed, with a weaker role for TTC.

Overall, the present study does not claim to fully eliminate the black-box problem in autonomous-driving control. Instead, it shows that learned brake behavior can remain substantially more interpretable when the learning problem is carefully structured and when the deployed hybrid behavior is analyzed with fallback-aware and quantitative tools. By grounding brake generation in semantically meaningful traffic-state features, anchoring the learned controller to a risk-aware reference policy, and analyzing deployed behavior through a high-fidelity surrogate-plus-SHAP pipeline, the study demonstrates a practical path toward more transparent, diagnosable, and scientifically interpretable learning-based safety control.

Several directions remain for future work. These include extending the analysis to additional maps, traffic seeds, and control regimes, studying temporal continuity more explicitly, comparing longitudinal and lateral interpretability under a unified framework, and exploring whether similar reference-to-distilled policy structures can support interpretable control in other safety-critical robotic tasks. More broadly, the results suggest that a useful middle ground exists between rigid hand-crafted safety logic and fully opaque learned policies, and that this middle ground may be especially valuable for the deployment of learning-based control in robotics and autonomous driving.

## Data Availability

The original contributions presented in this study are included in the article. Further inquiries can be directed to the corresponding author.
